# Genomic instability as a driver and suppressor of anti-tumor immunity

**DOI:** 10.3389/fimmu.2024.1462496

**Published:** 2024-10-11

**Authors:** Marta Requesens, Floris Foijer, Hans W. Nijman, Marco de Bruyn

**Affiliations:** ^1^ Department of Obstetrics and Gynecology, University Medical Center Groningen, University of Groningen, Groningen, Netherlands; ^2^ European Research Institute for the Biology of Ageing, University Medical Center Groningen, University of Groningen, Groningen, Netherlands

**Keywords:** genomic instability, MMRd, chromosomal instability, cGAS-STING, tumor-infiltrating lymphocytes, immune evasion

## Abstract

Genomic instability is a driver and accelerator of tumorigenesis and influences disease outcomes across cancer types. Although genomic instability has been associated with immune evasion and worsened disease prognosis, emerging evidence shows that genomic instability instigates pro-inflammatory signaling and enhances the immunogenicity of tumor cells, making them more susceptible to immune recognition. While this paradoxical role of genomic instability in cancer is complex and likely context-dependent, understanding it is essential for improving the success rates of cancer immunotherapy. In this review, we provide an overview of the underlying mechanisms that link genomic instability to pro-inflammatory signaling and increased immune surveillance in the context of cancer, as well as discuss how genomically unstable tumors evade the immune system. A better understanding of the molecular crosstalk between genomic instability, inflammatory signaling, and immune surveillance could guide the exploitation of immunotherapeutic vulnerabilities in cancer.

## 1 Introduction

Genomic instability, defined as an increased tendency of genomic alterations during subsequent cell divisions, is a hallmark of cancer and plays key roles in malignant transformation, cancer progression, and response to treatment (1, 2). The accumulation of mutations and genomic alterations can result in the activation of oncogenes, the inactivation of tumor suppressor genes, and the dysregulation of key cellular pathways, ultimately leading to transformation and uncontrolled cell proliferation. Through the acquisition of genetic diversity, genomic instability fuels tumor evolution and endows tumor cells with a survival advantage, enabling them to adapt to changing conditions and upon selective pressure (1, 2).

Nevertheless, genomic instability can have detrimental effects on cell viability. In addition to reducing tumor cell fitness through several cell-intrinsic mechanisms, genomic instability triggers a cascade of cell and non-cell autonomous inflammatory signaling pathways (3). This leads to the release of cytokines and chemokines to attract immune cells and the expression of immune-activating signals for the rapid elimination of DNA-damaged cells (4–6). Moreover, alterations in the DNA sequence or genomic rearrangements can lead to the generation of mutated proteins that will eventually be presented as neoantigens and activate T cells (7). Hence, immune surveillance is a critical barrier to tumorigenesis, especially in the context of genomic instability. As a result, cancer cells need to evolve and activate mechanisms to reduce immunogenicity and therefore avoid detection and elimination by the immune system.

Over the last few decades, our understanding of the mechanisms of immune evasion exploited by cancer has led to the development of effective immunotherapies, particularly immune checkpoint blockade (ICB) (8, 9). Even though pro-inflammatory signaling and genetic alterations have been correlated with response to ICB (8), durable response rates in genomically unstable tumors remain paradoxically low (10–14), limiting the use of those drugs and highlighting the need to develop novel complementary approaches. To do so, a thorough molecular understanding of the specific mechanisms of immune recognition and immune evasion of tumors with high genomic instability is crucial. This review focuses on the complex interplay between genomic instability and the immune system in the context of cancer. We summarize the main inflammatory consequences triggered by genomic instability, primarily through cGAS/STING activation, and how the different types of immune cells can recognize genomically unstable tumors. Finally, we discuss the main mechanisms of immune evasion exploited by tumors with defects in DNA repair and tumors displaying high levels of chromosomal instability (CIN) and resulting aneuploidy and their implications for cancer immunotherapy.

## 2 Cellular mechanisms of genomic instability in cancer

Genome stability is tightly monitored by several mechanisms, which include the DNA damage checkpoints, the DNA repair machinery, and mitotic checkpoints. Defects at any of these steps can result in genomic instability. Thus, genomic instability can present itself in multiple different levels ranging from single nucleotide point mutations to complex structural and numerical chromosomal abnormalities.

### 2.1 Defects in DNA repair

The DNA damage response (DDR) is a complex network of highly conserved pathways that have evolved to sense and repair various forms of DNA damage (3). The DDR comprises a number of mechanisms to repair various forms of damage including base excision repair (BER), single-strand break repair (SSBR), nucleotide excision repair (NER), mismatch repair (MMR), homologous recombination (HR), and nonhomologous end-joining (NHEJ). While deficiencies in any of those pathways can give rise to genomic damage and contribute to tumorigenesis (3, 15), here, we focus the role of MMR and HR on genomic instability.

#### 2.1.1 Defects in mismatch repair

**Figure 1:**
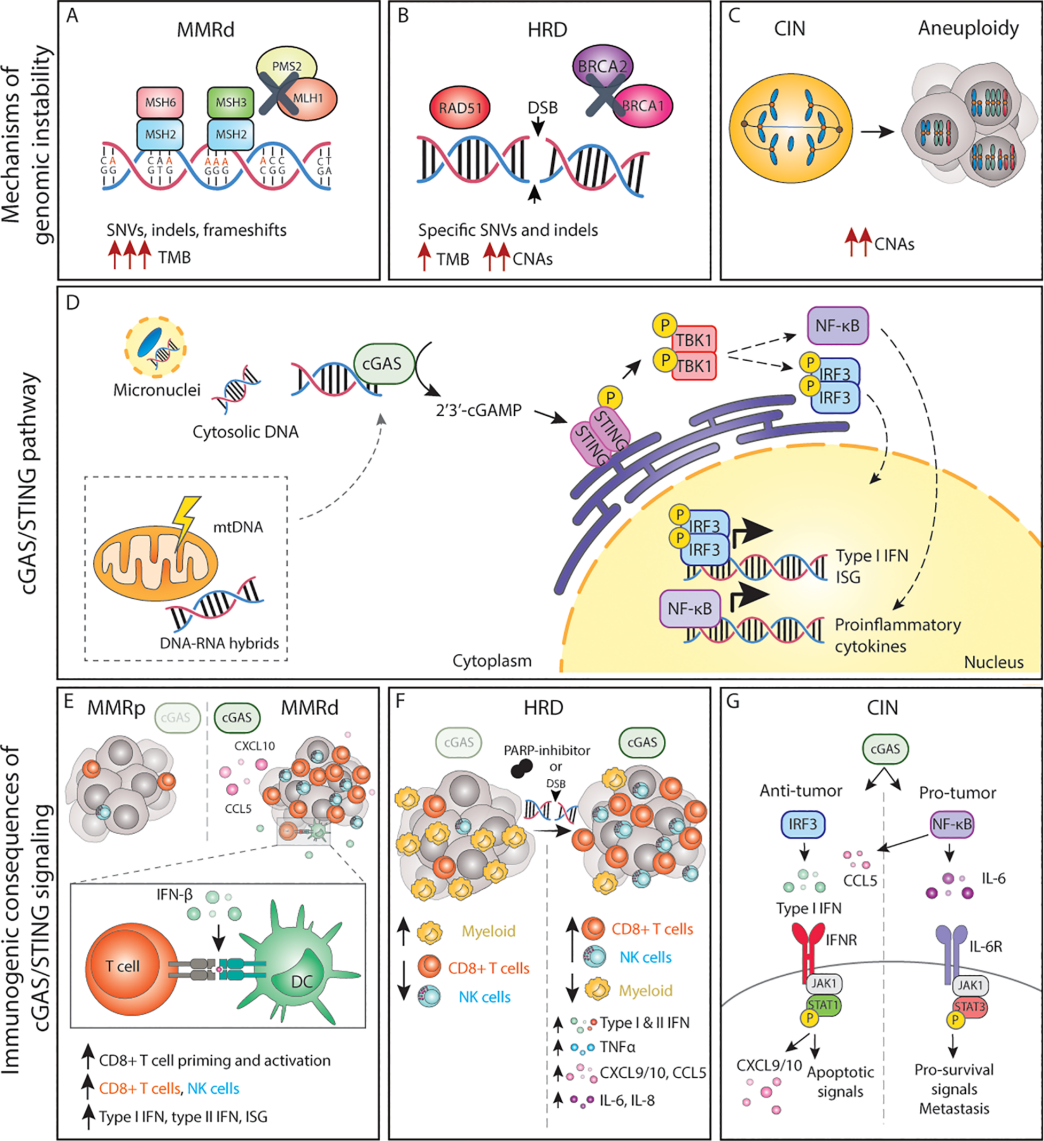
The cGAS/STING pathway and its inflammatory consequences in genomically unstable tumors. **(A-C)** Genomic instability can lead to the accumulation of dsDNA and other acid nucleic structures in the cytoplasm, which can activate cGAS. **(D)** Activation of cGAS either by nuclear DNA, micronuclei or other acid nucleic results in inflammatory signalling and expression of type I IFNs, ISG and NF-κB target genes. **(E)** In MMRd tumor, activation of cGAS results in increased CXCL10 and CCL5 production, which increase the number of tumor-infiltrating CD8+ T and NK cells as well as increased expression of IFN-β, which enhances DC-T cell interactions. **(F)** In HRD tumors, or tumors treated with DNA damaging agents such as PARP-inhibitors, activation of the cGAS pathways results in expression of the chemokines and cytokines, leading to a more inflamed TME with higher number of cytotoxic immune cells. **(G)** The cGAS/STING pathway in CIN/aneuploid cells has both pro and anti-tumor effects. The activation of the IRF3-type I IFN signalling axis results in immune surveillance and apoptotic signals, whereas NF-κB signalling mainly promotes IL-6/STAT3 pro-survival signals and enhance metastatic potential. MMRd, mismatch-repair deficient; MMRp, mismatch-repair proficient; MSH, mutS homolog; MLH1, mutL homolog 1; PMS1, PMS1 Homolog 2; SNV, single-nucleotide variations; indels, insertions-deletions; TMB, tumor-mutational burden; HRD, homologous-recombination deficiency; DSB, double-strand break; BRCA, breast cancer gene; CNAs, copy number alterations; CIN, chromosomal instability; mtDNA, mitochondrial DNA; cGAS, cyclic GMP-AMP synthase; STING, stimulator of interferon genes; TBK1, TANK-binding kinase 1; IRF, interferon regulatory factor; NF-κB, nuclear factor κB; ISG, interferon stimulated genes; CCL, C-C motif chemokine ligand; CXCL, C-X-C motif chemokine ligand; CD, cluster of differentiation; NK, natural killer; DC, dendritic cell; TNF, tumor necrosis factor; IL, interleukin; IFN, interferon; IFNR, interferon receptor; JAK1, janus kinase 1; STAT, signal transducer and activator of transcription; P, phosphorylation.

The MMR pathway recognizes and repairs base pair mismatches and small insertions and deletions loops (indels) that occurred during DNA replication (16). Mismatched base pairs and small indels up to 3 nucleotides can be recognized by the MSH2/MSH6 heterodimer whereas larger indels are recognized by the MSH2/MSH3 complex (17, 18). Once these heterodimers recognize the error, a second complex formed by MLH1/PMS2 is recruited and forms a tetrameric complex. Subsequently, exonuclease 1 (EXO1) is recruited, activated, and removes the newly synthesized DNA strand, leaving a DNA excision gap and a region with a single-stranded DNA (ssDNA). DNA polymerases and ligases subsequently synthesize and seal the new correct DNA strand, repairing the damage (16). Although the mutation rate during DNA replication is low and DNA polymerases have proofreading activity and can correct some of these errors, others escape proofreading and require the MMR system for repair (16) (**Figure 1A**).

Individuals with germline mutations in one or more MMR proteins (i.e. MLH1, MSH2, MSH6, PMS2), known as Lynch Syndrome (LS) patients, have a higher risk for developing early-onset cancers in multiple tissues (19–21). This indicates that loss of MMR functionality renders cells susceptible to malignant transformation (22, 23). In addition to LS patients, MMR is also frequently affected in sporadic (non-hereditary) cancers due to somatic mutations or loss of expression of one or more MMR proteins (24). The most common cause of MMR deficiency in human cancers is epigenetic silencing of the *MLH1* promoter (24–27). Sporadic MMR-deficient (MMRd) tumors are most commonly colorectal carcinoma (CRC) [15% of all CRC (24)] and endometrial carcinoma (EC) [30% of all EC (28)] but can also arise in several other tissue types including stomach, brain (mainly glioblastoma), ovarian, and pancreas, among others (29–31). The reasons why certain tissues develop MMRd tumors more frequently than others are yet unknown.

Loss of function of one or more proteins of the MMR system results in failure to repair replication errors and thus persistence of mutations throughout the genome, particularly in regions of repetitive DNA sequences, known as microsatellite (MS) regions (32, 33). Errors in MS regions most commonly arise from polymerase slippage. This phenomenon is known as microsatellite instability (MSI) and is a hallmark of MMRd tumors. When MSI occurs in coding regions, it can lead to alterations in open reading frames, yielding functionally inactive proteins, including proto-oncogenes and tumor suppressor genes (31, 34). In addition to MSI, MMRd results in a high rate of single nucleotide variation (SNVs) and large numbers of frameshifts that result in indels. Therefore, MMRd cancers are typically considered hypermutated tumors that display a substantial tumor mutational burden (TMB) and a high number of predicted neoantigens (24, 31, 35, 36) (**Figure 1A**).

Besides MMRd cancers, there is a subset of tumors that harbor mutations in the exonuclease domain of the catalytic subunit of the DNA polymerase epsilon (POLE) or the polymerase delta (POLD1), resulting in loss or defective proofreading (37, 38). These tumors can also arise from a germline or somatic mutation in *POLD1* or *POLE* genes, with *POLE* mutations in 1%-10% of all CRC and EC cases (24, 38–40). Even though these tumors have relatively low MSI rates and display different MSI signatures than MMRd (41), POLE-mutant (POLE-mut) tumors have dramatically higher rates of SNVs and are therefore considered ultra-mutated tumors (37, 42). While MSI is high in MMRd tumors and SNV rates are high in both groups, in general, MMRd and POLE-mut tumors display relatively stable karyotypes and low numbers of copy number alterations (CNAs) (43).

#### 2.1.2 Defects in homologous recombination

HR is the mechanism primarily responsible for repairing double-strand breaks (DSBs) in DNA during the S and G2 phases of the cell cycle. Upon recognition of DSBs, the 5’ end of the break is resected, creating a ssDNA region that serves as a template for the repair. The exposed ssDNA regions are protected from degradation. BRCA1/BRCA2 mediate recruitment at the DSB site and facilitate the repair. After strand invasion by RAD51, a complex DNA structure is formed (D-loop) followed by DNA synthesis. The newly synthesized DNA can be directly ligated to the original DNA, or alternatively, a holiday junction (HJ) is formed that needs to be further resolved or dissolved by specific proteins to complete the repair (44).

Germline mutations in HR genes including *BRCA1* or *BRCA2* predispose to early-onset breast and ovarian cancer (45), indicating that the genomic instability caused by HR defects drives malignant transformation. In addition, somatic alterations in *BRCA1/2* and other HR-related genes such as *ATM* or *RAD51* are prevalent among several cancer types including ovarian, breast, pancreas, and prostate, among others (46). A defective or compromised HR system leads to unrepaired DSBs, accumulation of DNA lesions, and collapsed replication forks resulting in complex genomic rearrangements and increased susceptibility to accumulation of mutations. In line with this, the genomes of HR-deficient (HRD) cancers are generally characterized by complex CNAs and are highly aneuploid, but may also display specific base substitutions and indels, thus yielding an intermediate TMB (46–49). In fact, cancers with mutations in HR genes have a higher TMB and higher number of predicted neoantigens in comparison to their HR-proficient counterparts (48, 49) (**Figure 1B**).

### 2.2 Chromosomal instability

CIN is defined as persisting errors in chromosome segregation during mitosis, leading to numerical and/or structural chromosomal abnormalities in the resulting daughter cells (50). Aneuploidy is the result of CIN, and it is a state in which cells harbor alterations in the chromosomes. CIN can yield numerical aneuploidy i.e. an abnormal number of whole chromosomes, as well as structural aneuploidy, when chromosome fragments display CNAs due to unbalanced translocations, inversions, or deletions (50) (**Figure 1C**). Faithful chromosome segregation relies on the structural integrity of the microtubule spindle machinery and the spindle assembly checkpoint (SAC). The various processes that can lead to aneuploidy have been extensively described elsewhere and include but are not limited to centrosome amplifications, telomere dysfunction, deregulation of genes involved in the SAC, loss of cohesion, altered microtubule polymerization rates or abnormal kinetochore-microtubule dynamics (51).

Numerical and structural aneuploidies will lead to unbalanced gene expression, thus disrupting normal cellular processes, and being detrimental to the viability of healthy cells. Furthermore, CIN and aneuploidy promote proteotoxic stress, replication stress, and increased DNA damage, which often leads to cell cycle arrest or cell death (52). Even though CIN and aneuploidy are not well tolerated in healthy tissues (53, 54), over 80% of all human solid tumors display chromosomal abnormalities (55). Furthermore, CIN and/or aneuploidy are associated with (multi) therapy resistance (56–58), immune evasion (59–61), metastasis (62, 63), and thus an overall poor patient prognosis (64, 65). This can likely be explained by the fact that ongoing CIN will promote the generation of new karyotypes during tumorigenesis, thus driving cancer cell evolution and intratumor karyotype heterogeneity (66). Therefore, tumors with high CIN rates will have a large variety of distinct karyotypes and upon (new) selective pressure, cells with specific chromosome combinations may hold a survival advantage and can be selected for.

## 3 Inflammatory signaling in genomically unstable cancers

DNA is normally localized in the nucleus and mitochondria. Genomic instability can lead to DNA being exposed into the cytoplasm for instance as a result of stalled replication (67), mitochondrial damage (68), or by mis-segregated chromosomes and chromosome fragments that yield micronuclei (69). Micronuclei are prone to rupture, eventually exposing the genomic DNA to the cytoplasm (70). Cells are equipped with pattern recognition receptors (PRR) that sense loss of cellular homeostasis, including mislocalized or aberrant DNA and RNA structures. Cytoplasmic nucleic acid sensors are PRRs that signal foreign and host-derived DNA and RNA and when activated, initiate cell autonomous and cell extrinsic defense mechanisms (71).

A well-known cytoplasmic DNA sensor is the cyclic GMP-AMP synthase (cGAS), which recognizes and responds to cytosolic double-stranded DNA (dsDNA) in a DNA-sequence independent manner, serving as a ubiquitous DNA sensor (72, 73). Besides dsDNA originating from the nucleus, other forms of nucleic acid are also able to activate cGAS such as oxidized self-DNA (74), mitochondrial DNA (mtDNA) (75, 76) and, as more recently shown, DNA-RNA hybrids (77). These and other mislocalized nucleic acid structures such as endogenous dsRNA, ssRNA, or mitochondria dsRNA can also be recognized by other cytosolic sensors including RIG-I, MDA5, AIM2, IFI16, and TLRs, among others [reviewed elsewhere (71)]. Here, we will focus on the role of cGAS/STING activation in cancers with high genomic instability, including tumors with DNA repair defects and tumors displaying CIN/aneuploidy.

### 3.1 The cGAS-STING pathway

cGAS binding to cytoplasmic dsDNA leads to its enzymatic activation and subsequent production of cGAMP, a second messenger molecule and a potent agonist of Stimulator of Interferon Genes (STING) (78). Activated STING recruits TANK-binding kinase 1 (TBK1) to initiate further downstream signaling that ultimately leads to translocation of interferon regulatory factor 3 (IRF3) dimers to the nucleus and the transcription of anti-viral-like gene programs such as type I interferons (IFN) as well as pro-apoptotic genes (79) (**Figure 1D**). Type I IFNs in turn activate interferon-stimulated genes (ISG) in an autocrine and paracrine manner, including genes in the janus kinase (JAK)/Signal transducer and activator of transcription (STAT) pathway. Whereas JAK/STAT1 signaling plays a key role in the induction of cell death and anti-tumor immunity, JAK/STAT3 signaling has anti-apoptotic effects and promotes cell growth (80). Parallel to IRF3, STING can also activate both the canonical (RELA-p50) and non-canonical (RELB-p52) Nuclear Factor-κB (NF-κB) to induce other inflammatory gene programs (79, 81) (**Figure 1D**). These signaling cascades do not operate independently but rather constitute a complex signaling network displaying multiple levels of crosstalk and feedback control that result in a pleiotropic response (81). Yet, the magnitude of this response, its dynamics, and the interconnections between these pathways are still poorly understood.

Over the last decade, the cGAS/STING pathway has received significant attention in the cancer field as it plays a pivotal role in mounting anti-tumor responses both in a tumor cell autonomous and non-autonomous manner. Chronic activation of cGAS/STING can result in the secretion of soluble factors collectively known as the senescence-associated secretory phenotype (SASP), which can restrict malignant growth (82–84). Additionally, cGAS/STING-induced pro-inflammatory chemokines and cytokines have a wide range of immune-stimulatory effects shown to induce potent anti-tumor responses (85). Importantly, intrinsic tumor STING expression helps in immune-mediated control of metastatic quiescent cancer cells (86). The cGAS/STING pathway can also coordinate multicellular immune responses through extracellular signaling of cGAMP, amplifying pro-inflammatory signaling (87). cGAMP intracellular communication can be mediated by cell-to-cell junctions that directly connect the cytosol of adjacent cells or by cGAMP-containing exosomes secreted in the extracellular space that can be taken up by recipient cells. In the context of anti-tumor immunity, cancer-derived cGAMP or cancer-derived DNA have been shown to activate dendritic cells and macrophages, which in turn respond by producing type I IFN to enhance CD8+ T cell anti-tumor activity (88–90). Similarly, release of damaged DNA resulting from telomere stress also results in enhanced immune responses (91). This is in line with the observation that NK cell cytotoxicity against tumor cells requires STING expression by host cells and cGAS expression by cancer cells (90). Exploring whether extracellular cGAS/cGAMP signaling is particularly relevant in tumor cells with high genomic instability remains to be investigated.

Conversely, other work has shown a tumor-promoting role for the cGAS/STING pathway. In preclinical models, chronic inflammation induced by 7,12-dimethylbenz(a)anthracene (DMBA) was shown to promote skin tumorigenesis in a STING-dependent manner through IL-6 expression (92). Further studies have shown that cGAS/STING signaling can promote tumor cell survival in an autocrine and paracrine manner (93, 94). How these opposite roles are mechanistically regulated remains unclear, but their dynamics likely determine the ultimate output of cGAS/STING, thereby being very context dependent.

The inflammatory consequences and anti- and pro-tumor effects triggered by sensing of cytosolic DNA in tumors with defects in DNA repair and chromosome imbalances are described in more detail below.

#### 3.1.1 cGAS/STING-driven inflammatory signaling by deficient MMR

Recent work has shown that MMRd human tumors, as well as MMR-deficient engineered cell lines, display enhanced type I and type II IFN signatures in comparison to their MMR-proficient (MMRp) counterparts and that this is mediated by activation of the cGAS/STING pathway (95–97). Mechanistically, cells with an inactivated *MLH1* gene accumulate cytosolic DNA by dysregulation of MLH1-dependent EXO1 function, which causes unrestrained DNA hyperexcision and aberrant DNA breaks (95). As a result, MLH1 KO cells accumulate cytoplasmic DNA, which eventually results in the expression of ISG15 and IFN-β in a cGAS and STING-dependent manner (97). Further *in vitro* and *in vivo* studies have demonstrated that cancer-cell intrinsic cGAS activation and subsequent type I IFN expression is necessary for optimal CD8+ T cell priming. While defects in MMR reduced *in vivo* tumor growth in comparison to wild-type tumor cells, concomitant deletion of tumor cGAS, STING, or IFNAR1 rescued these growth defects, indicating a key role of cGAS/STING-type I IFN axis in immune surveillance (97). These and other studies have reported the importance of type I IFN signaling in dendritic cell (DC) -T cell interactions (98–101) (**Figure 1E**).

However, type I IFN is not the only factor produced upon cGAS engagement. CCL5 and CXCL10 chemokines are also upregulated in response to cGAS/STING. Indeed, anti-tumor immunity in MMRd cancers also depends on the magnitude of cGAS/STING activation and subsequent expression of CCL5 and CXCL10 in the tumor microenvironment (TME), which influences CD8+ T and NK cell infiltration (96, 102) (**Figure 1E**). *In vivo*, blockade of the CXCR3-CXCL10 interaction with monoclonal antibodies dampened the infiltration of immune cells into the tumors and accelerated tumor growth (96). In comparison to MMRp CRCs, gene and protein expression levels of both cGAS and STING are significantly higher in MMRd, and such tumors display higher CXCL10, CCL5, and CD8+ T effector gene signatures (103, 104). In fact, both cGAS and STING expression correlates with an increase in CD8+ but not CD4+ T cell numbers (104). Although a similar phenotype with accumulation of cytoplasmic DNA and enhanced expression of type I IFN, ISG15, CXCL10, and CCL5 has been shown for MSH2 KO cell lines (96), the mechanism of cGAS activation upon MSH2 deficiency remains to be determined. Altogether, these data underscore the importance of the cGAS/STING-driven inflammatory phenotype in MMRd tumors for optimal anti-tumor immunity.

#### 3.1.2 cGAS/STING in cancers with HR defects

In the context of HR, loss, inhibition or mutations in *BRCA1/2* lead to the generation of cGAS-positive micronuclei and induction of cGAS/STING signaling which results in the expression pro-inflammatory type I and type II IFNs, NF-κB dependent TNFα activation, as well as expression of CXCL10, CXCL9, CCL5, IL-6, and IL-8 (105–109), extensively reviewed elsewhere (110) (**Figure 1F**). Similar effects are observed in cells with alterations in other HR genes, defective DDR, or upon DNA-damaging agents (109, 111, 112) (**Figure 1F**). For instance, defects in *ATM* also result in cytoplasmic accumulation of STING, its subsequent activation, and the initiation of a type I IFN response (113).

In breast cancer, higher cGAS/STING scores are associated with higher genomic instability, which in turn correlate with overall higher infiltration of immune cells (114). *BRCA1/2* mutations are indeed often associated with an increased number of tumor-infiltrating lymphocytes in different cancer types (108, 109, 115, 116) and tumor-associated inflammation in BRCA1-mutant breast cancer has significant positive prognostic value (108, 117). In fact, tumors with HR mutations are generally associated with a better patient prognosis than their wild-type counterparts, beyond BRCA mutations (118). Therapeutically, HR deficiency sensitizes tumors to DNA-damaging agents and poly(ADP-ribose) polymerase (PARP) inhibitors. Studies using mouse models of breast and ovarian cancer have revealed that the efficacy of PARP inhibition in BRCA-deficient tumors depends on the activation of the cGAS/STING pathway. Mechanistically, PARP inhibitors increase expression of type I IFN in the TME in a cGAS/STING-dependent manner, which in turn mediates recruitment and activation of effector T cells (119, 120) and NK cells while reducing the number of myeloid cells (121) (**Figure 1F**). Overall, cGAS/STING activation appears to be relevant for enhancing anti-tumor immunity in the context of HRD cancers both at baseline and upon DNA-damaging therapies.

#### 3.1.3 cGAS/STING in tumors displaying CIN or aneuploidy

Chromosome mis-segregation events during mitosis can lead whole chromosome and chromosome fragments to end up in the cytoplasm as micronuclei. Rupture of the micronucleus membrane exposes the DNA in the cytoplasm, activating the cGAS/STING pathway (122, 123). Mackenzie and colleagues demonstrated that cGAS quickly localizes to such micronuclei and that ISG genes including CCL5 and CXCL10 are induced almost exclusively in micronuclei-positive cells (69). Indeed, drug-induced CIN triggers type I IFN, canonical and non-canonical NF-κB and eventually STAT1 and STAT3 signaling in an autocrine and paracrine manner (62, 93, 124, 125), extensively reviewed elsewhere (126) (**Figure 1G**). While micronuclei appear to be an important hub for cGAS activation, recent work suggests that the formation of chromatin bridges prior to micronucleus formation is required to activate the cGAS/STING pathway (127).

In addition to cells exhibiting ongoing CIN, stable aneuploid cells also display an inflammatory phenotype. In contrast to euploid cells, cells with trisomy 21 show a constitutive IFN signature and enhanced ISG response upon IFN stimulation (128). In fact, there seems to be a general inflammatory response to the presence of any extra chromosome as the cGAS/STING/TBK1/IRF3/STAT1 axis was found to be constitutively active in cells with any trisomy, presumably due to accumulation of cytoplasmic dsDNA (129). Furthermore, cells with complex aneuploid karyotypes show transcriptome signatures that include type I IFN response, allograft rejection, antigen processing and presentation as well as activation of NF-κB signaling (6). Another inflammatory characteristic of CIN and aneuploid cells is increased expression of SASP-like cytokines, such as IL-1β, CXCL8, CCL2, CCL27, and TNFs (130). Together these pathways may sustain a chronic inflammatory phenotype, potentially resulting from cGAS/STING engagement, which might aid in the clearance of aneuploid and cells displaying CIN by the immune system.

Conversely, recent work has shown that chronic activation of cGAS/STING favors tumor growth, specifically in the context of cancers displaying high levels of CIN. For instance, cGAS/STING was shown to contribute to increased cancer cell survival through autocrine IL-6/STAT3 signaling in cells with induced CIN phenotypes (93) (**Figure 1G**). Furthermore, cytosolic DNA was shown to promote metastasis in a cell-autonomous manner via STING/non-canonical NF-κB activation (62) as well as in a non-autonomous cell manner via STING-dependent modulation of the immune system (131). Interestingly, cGAS/STING can lead to the activation of non-canonical NF-κB signaling without altering IFN signaling, indicating that cancer cells can eschew type I IFN signaling while benefiting from cGAS/STING-induced pro-tumor inflammation (62, 131). Collectively, this work demonstrates that cGAS/STING activation has both anti and pro-tumorigenic effects in a context-dependent manner. Acute cGAS/STING activation may lead to cell death, cell cycle arrest, and immune surveillance, whereas chronic cGAS/STING stimulation may ultimately promote survival and initiation of metastasis.

#### 3.1.4 Mitochondrial genomic instability and cGAS/STING signaling

MtDNA is also replicated over cell divisions and mitochondria have multiple replication and repair mechanisms to maintain mtDNA integrity (132–135). Similar to nuclear DNA, defects in mtDNA repair mechanisms have been associated with increased malignant potential (136). Furthermore, many cancer types are thought to harbor driver mutations in the mtDNA (137), highlighting the role of mitochondrial genomic instability in tumorigenesis. Beyond nucleotide mutations, mtDNA is vulnerable to damage due to its proximity to the mitochondrial electron transport chain, where reactive oxygen species (ROS) are generated as byproducts of cellular respiration. Under cellular stress, mitochondrial damage or mitochondrial dysfunction, mtDNA or oxidized mtDNA (ox-mtDNA) can exit the mitochondria and activate cGAS/STING. This triggers inflammatory signaling including expression of ISGs and the production of type I IFNs and chemokines such as CXCL10 (68, 138, 139), which may have intrinsic and extrinsic effects. Additionally, mtDNA has been shown to contribute to the SASP via activation of the cGAS/STING (140) and extracellular mtDNA release by senescent cells contributes to an immunosuppressive TME (141).

It is important to notice that, beyond cGAS/STING, mtDNA (and mtRNA) can activate a wide range of other inflammation pathways including NLRP3 inflammasome, RIG-I, TLR9, ZBP1 or IFI16, described in more detail elsewhere (142). Despite this evidence, the specific consequences of mtDNA-driven inflammation within tumors, as well as its impact on the overall immune environment and response to immunotherapy are yet to be fully understood. Furthermore, it would be interesting to investigate whether tumors with high nuclear genomic instability (e.g. CIN) also exhibit increased mitochondrial genomic instability.

Next to MMRd, HRD or CIN, it would be interesting to understand how other forms of genomic damage or genomic alterations such as centromere defects (143), DNA damage at telomeres (144), chromotripsis or toroidal nuclei affect cGAS/STING-driven inflammation and immune responses and how these pathways intersect with each other.

## 4 Immunogenicity of genomically unstable cancers

Tumor immunogenicity is the ability of tumor cells to induce a strong immune response, which can greatly vary between cancer types and individuals. Key determinants of tumor immunogenicity include but are not limited to the specific oncogenic drivers (145–147), the TMB (7) and/or the intrinsic inflammatory phenotype of the tumor cells (148). How the different types of genomic instability trigger inflammatory signaling and immune surveillance and how these pathways are intertwined in tumors with high genomic instability is described in more detail below.

### 4.1 Cancers with MMR defects

The genomic instability resulting from MMR deficiency significantly impacts the TME, the progression of the disease and the response to (immuno)therapy. In comparison to their proficient counterparts, MMRd tumors have been consistently reported to have an inflamed microenvironment with high infiltration of cytotoxic immune cells, which is correlated with an overall better prognosis and, importantly, better response to immunotherapy (149–151).

In the last decade, transcriptional profiles of bulk tumor samples and single cells from large cohorts of CRCs allowed for the classification of these tumors in 4 consensus molecular subgroups (CMS) based on their cellular composition (43, 152). The first CMS was enriched for MMRd/MSI-high tumors displaying high TMB and low somatic CNAs (SCNAs). In comparison to the 3 other CMS, the MMRd-enriched subgroup shows significantly higher infiltration scores of cytotoxic lymphocytes and Th1 cells, low infiltration of regulatory T cells, and high expression of genes encoding for cytotoxic T cell attracting chemokines, Th1 cytokines, and other cytokines and chemokines involved in anti-tumor immunity such as IFNs, CXCL13 and IL15 (43, 152). These findings were further substantiated using the CIBERSORT algorithm in a more recent study (153) and observed in other MMRd tumor types, including EC (39, 154, 155). Furthermore, MMRd tumors have been shown to generate systemic robust immune responses (156). The diverse factors contributing to increased immunogenicity and immune activation in MMRd tumors are reviewed below.

#### 4.1.1 TMB, neoantigens and CD8+ T cells

**Figure 2:**
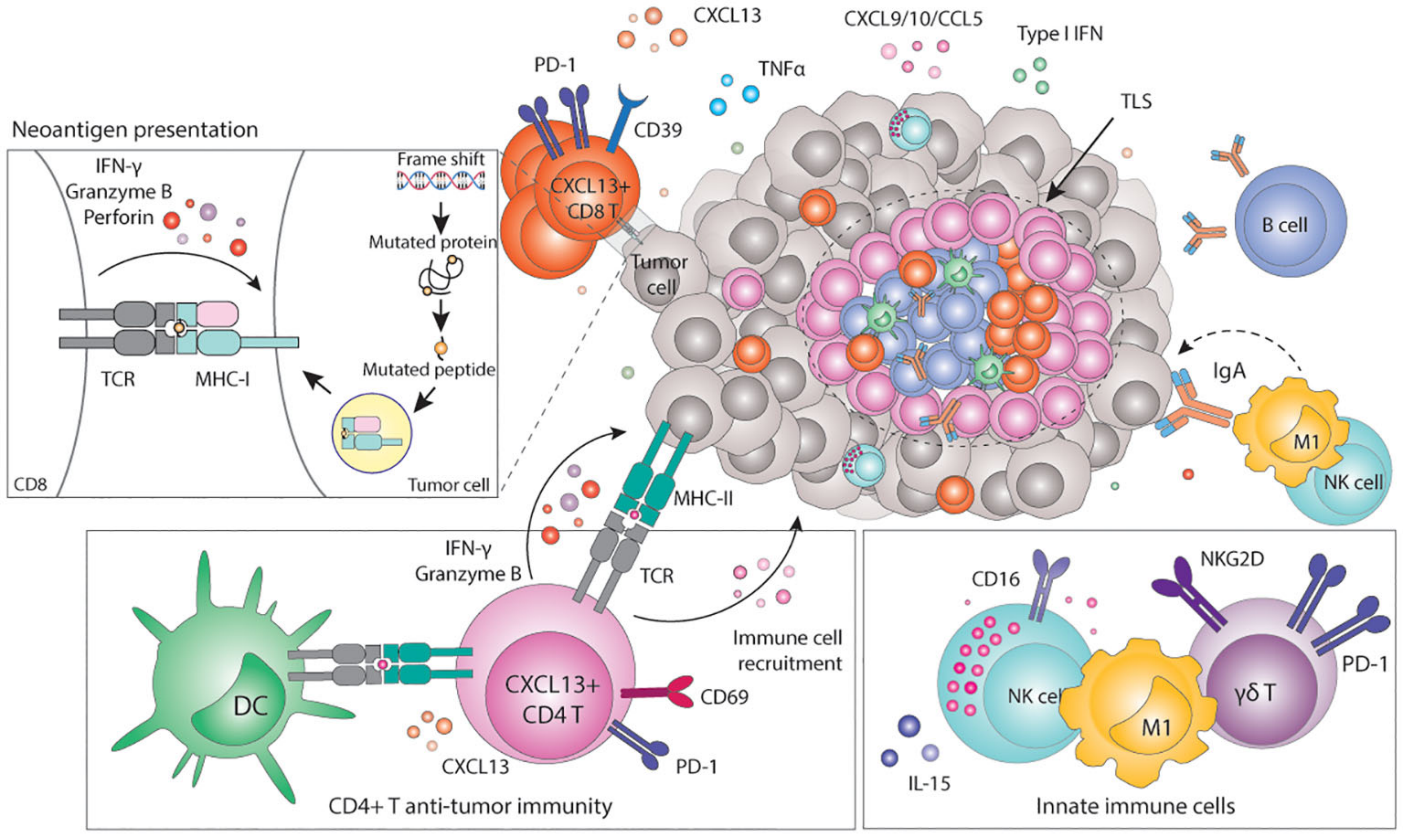
Immunogenicity of MMRd tumors. Frameshifts and SNV eventually result in the expression of neoantigens loaded in the MHC-I complex. Recognition of neoantigens activates CD8+ T cells and subsequent expression of cytotoxic molecules including granzyme B, IFN-γ or perforin, leading to the elimination of tumor cells. Tumor cells can also express neoantigens loaded on the MHC-II, which can be recognized by CD4+ T cells and initiate a cytotoxic response. In parallel, CD4+ T cells can also express pro-inflammatory cytokines and chemokines to recruit myeloid and NK cells that contribute to enhance anti-tumor immunity. Innate γδ T cells can target tumor cells via the NKG2D ligand-receptor interaction. High TMB correlates with the presence of TLSs in the tumor, which are highly organized hubs of immune cells that shape both adaptive and humoral immune responses. In particular, TLSs contain B-cell producing antibodies that may bind tumor antigens and trigger antibody-dependent cytotoxicity (ADCC). MHC, Major histocompatibility complex; TCR, T cell receptor; CD, cluster of differentiation; PD-1, programmed-death 1; TLS, tertiary lymphoid structure; Ig, immunoglobulin; CXCL, C-X-C motif chemokine ligand; CCL, C-C motif chemokine ligand; TNF, tumor necrosis factor; IFN, interferon; M1, macrophage type 1; NK, natural killer; IL, interleukin; NKG2D, natural killer group 2 D.

MMRd tumors accumulate many mutations in the DNA sequence, quantified as the TMB (157). This may result in the production of mutated proteins that can eventually be presented as (neo)antigens loaded in the Major Histocompatibility Class-I (MHC-I) complex. MHC-I complexes are expressed on the surface of all nucleated cells and continuously present the host proteome in the form of peptides to CD8+ T cells, thus playing a critical role in the adaptive immune system (158). CD8+ T cells are MHC-I-restricted, and therefore can only recognize peptides loaded in the MHC-I. The MHC-I-peptide complexes are scanned by CD8+ T cells, which mount potent, specific, and long-lasting immune responses when non-self-peptides are identified (159). Several studies have shown a very strong positive correlation between TMB, CD3+, or CD8+ T cell infiltration and favorable prognosis in many cancer types, particularly in MMRd ones (103, 160–163). In fact, the number of frameshift mutations and the number of predicted neoantigens correlates with the density of infiltrating CD8+ T cells and lymphocyte score, respectively (163, 164) (**Figure 2**). Not only do MMRd tumors contain higher numbers of tumor-infiltrating T cells, but these T cells also have higher expression of activation and cytotoxic markers such as IL-2Rα (165), granzyme B (165, 166), perforin (167), PD-1 and IFN-γ (153, 162, 168, 169). Indeed, single-cell RNA-sequencing experiments revealed that the T cell compartment is the key difference between the immune composition of MMRd and MMRp CRCs (170). In line with previous observations, tumor infiltrating T cells in MMRd tumors exhibited gene signatures related to cytotoxicity (granulysin, granzymes, and perforin) as well as activation (PD-1) consistent with chronic stimulation. Furthermore, MMRd tumors contain abundant T cells with a strong CXCL13+ signature, suggesting effector tumor-specific T cells (170, 171). Similarly, MMRd ECs are enriched in CD8+ T cells expressing high levels of PD-1, CD39, TIM-3 and CXCL13, which define a population of tumor-reactive T cells that was also positively correlated with the level of the TMB (160) (**Figure 2**).

The current dogma is that the high TMB drives the inflammatory and immune activated phenotype. Indeed, pre-clinical studies have shown that defects in DNA repair trigger neoantigen generation and promote immune surveillance of tumors *in vivo* in a CD8-dependent manner (7, 150). Moreover, the degree of TMB within MMRd tumors affects their growth rate and response to ICB (150). More interestingly, in preclinical MMR heterogeneous tumors, the immunogenicity of MMRd cells was shown to drive the elimination of MMRp cells within the same local microenvironment (172). Clinically, TMB or surrogate markers such as MMRd are often one of the strongest predictors of response to ICB across cancer types (151, 173–175) and the degree of TMB and MSI status (MSI-high vs MSI-low) predicts long-term benefit to ICB (153). However, despite being a relevant factor, increasing evidence suggests that TMB is necessary but not sufficient to fully explain the strong anti-tumor immunity towards cancers with a hypermutated phenotype (176). In studies using murine lung and colon cancer models, MMRd and the resulting high TMB were not sufficient to increase the immunogenicity nor sensitivity of tumors to ICB. Instead, the presence of clonal neoantigens was shown to be more critical for effective T cell responses (177). Furthermore, the fact that cGAS or STING-deficient MMRd cell lines grow faster than the cGAS or STING-proficient MMRd counterparts in immunocompetent mice supports the idea that anti-tumor immunity does not solely rely on the expression of neoantigens (97). These observations underscore the importance of (cGAS/STING) inflammatory signaling and antigen-independent mechanisms of immune recognition and activation in cancers with MMR defects.

#### 4.1.2 CD4+ T cell anti-tumor immunity

In addition to the high infiltration of cytotoxic CD8+ T cells, MMRd tumors also show significant infiltration of CD4+ T cells compared to their MMRp counterparts. Premalignant polyp lesions of LS patients are densely infiltrated by IFN-γ-expressing CD4+ T cells, suggesting a role for CD4+ T cell recruitment and activation early during tumorigenesis (167). Similarly, significant CD4+ T cell infiltration has been observed in MMRd carcinomas, though in variable proportions. These effects also differ per tumor type: while in CRC CD8+ T cells are typically the dominant T cell type, MMRd ECs have higher densities of CD4+ T cells (160). CD4+ T cells more often localize in the invasive front and express higher levels of PD-1 and IFN-γ compared to MMRp tumors (168). Flow cytometry analysis showed enrichment of IFN-β-expressing CD4+ T cells in MMRd CRCs (178). Furthermore, these cells were positive for CXCL13 and co-express high levels of PD-1, CD27, CD39 and Ki67 (178), suggesting tumor reactivity (179). Indeed, PD-1^high^ CD4+ T cells isolated from MMRd ECs co-expressed other activation markers such as CD38, HLA-DR, ICOS, BCL6, CXCL13 and KI67, were proven to be tumor-reactive T cells and correlated with improved prognosis in ECs with high TMB (160). Interestingly, CD4+ T cells can drive strong anti-tumor immunity upon ICB in preclinical models of MMRd tumors with defective or low expression of MHC-I, which cannot be recognized by CD8+ T cells (180, 181). Under these circumstances, CD4+ T cells show high expression of the activation markers PD-1 and CD69, and cytotoxic molecules such as granzyme B (180) (**Figure 2**). However, the mechanisms of CD4+ T cell mediated control of MMRd MHC-I-deficient tumors remain to be fully elucidated.

Emerging evidence in different models indicates that CD4+ T cells may play a role in anti-tumor immunity, distinct from their conventional function as helpers and regulators of cytotoxic CD8+ T cells (182). A subset of CD4+ T cells can acquire cytolytic function towards MHC-II-expressing tumor cells (183, 184). MHC-II is generally expressed by professional antigen presenting cells (APCs) and is used to present extracellular peptides from pathogens or tumors to CD4+ T cells. In contrast to CD8+ T cells, CD4+ T cells are MHC-II-restricted and therefore, they can only recognize peptides loaded onto the MHC-II complex. It has been reported that, under certain conditions, tumor cells can also express MHC-II. Because of the high TMB of MMRd tumors, it could be that neoantigens are also presented in the context of MHC-II, activating potent CD4+ T cell anti-tumor immunity. Indeed, CD4+ T cells isolated from MMRd ECs showed MHC-II-restricted tumor reactivity to autologous tumor cells (160)(**Figure 2**). Other work has shown that CD4+ T cells can target tumor cells independently of MHC-II by mobilizing or activating myeloid cells and NK cells (182, 185, 186). Overall, this data suggests that CD4+ T cells have a prominent cytotoxic role in anti-tumor immunity in MMRd tumors (including MHC-I proficient ones) more than previously anticipated.

#### 4.1.3 Innate immune cells

As opposed to the adaptive immune system, the role of innate immunity in MMRd tumors remains largely unexplored. Increased infiltration of innate γδ-like T cells has been reported in some MMRd CRC cohorts (170, 187) and these cells express higher levels of PD-1 in comparison to MMRp tumors (170). Analysis of MMRd tumors treated with a combination of anti-PD-1 and anti-CTLA-4 showed a role for γδ-T cells in targeting MMRd tumor cells with defective antigen presentation potentially via de NKG2D/NKG2DL ligand-receptor interaction (188). Other studies have found a positive correlation between MMRd status and infiltration of both macrophages and NK cells (153, 187). Activated NK cell gene signatures were also enriched in MMRd tumors (153) and circulating CD16+ NK cells showed strong upregulation of cytotoxicity and activation genes in EC MMRd patients responding to ICB (156). In comparison to non-responders, NK cells from responding patients express higher levels of granzyme A, EOMES, and TNF-mediated signaling molecules and were associated with longer survival (156). Anti-tumor M1-like macrophages were shown to be predominant in MMRd tumors and had significant prognostic value (187). A more recent study showed that whereas the abundance of monocytes and macrophages is similar between MMRp and MMRd CRC, macrophages from MMRd tumors express more inflammatory factors, chemokines and immune-activating alarmins than their MMRp counterparts (170), potentially contributing to an immunoreactive microenvironment. However, data remains scarce and heterogeneous and the precise functional contribution of innate immune cells in anti-tumor immunity and response to immunotherapy in the context of MMRd tumors remains to be elucidated.

#### 4.1.4 Tertiary lymphoid structures (TLSs) and B cells

An interesting observation is the positive correlation between neoantigen burden, TMB, and the presence of tertiary lymphoid structures (TLSs) (189). TLSs are highly organized ectopic lymphoid structures, mainly comprised of B and T cells, that provide niches for multiple crosstalk between immune cells. TLSs are thought to shape antigen-specific immune responses, clonal expansion, and increase cytokine-mediated signaling (190, 191). The presence of TLSs is a prognostic factor in several cancer types (192–199), as it is associated with a protective immunity and correlates with favorable outcomes both in primary and metastatic disease (190, 195, 197, 198, 200, 201).

Using a TLS gene signature, Lin and colleagues proposed that neoantigen load and the total TMB correlate with the presence of TLSs in multiple cancer types, including ECs (189). In line with these findings, TLSs were more frequent in MMRd and POLE-mut ECs, and the presence of 1 or more TLSs in these tumor types was a beneficial prognostic value (189, 202). While the mechanism underlying this phenomenon is thus far poorly understood, a contributing factor seems to be the increased presence of B cells and CXCL13-producing T cells in high TMB tumors (203). CXCL13 is a B-cell chemoattractant involved in lymphoid neogenesis and B cell differentiation (204). Workel and colleagues showed that CXCL13+ T cells are enriched in tumors with a high TMB, which correlates with the presence of TLSs (203). In agreement with this, MMRd CRCs display strong TLS signatures and have a significant presence of CXCL13-expressing T cells throughout the tumor and CXCL13-expressing follicular DCs in TLSs (43, 170). Increased presence of TLSs in tumors with a high TMB may also help improve humoral responses. Indeed, compared to other EC subtypes, only MMRd ECs showed an increased abundance of B cell-derived IgA antibodies in the TME. These antibodies were further shown to increase the expression of immunostimulatory cytokines such as TNF and IFN, overall increasing anti-tumor immune responses (199)(**Figure 2**). In other cancer types, IgA antibodies bind polymeric IgA receptors on tumor cells, which in turn enhances tumor targeting by myeloid and T cells (205). Nevertheless, how exactly the TMB instigates immune cells to drive the formation of TLSs and how this contributes to improved survival remains to be fully elucidated.

### 4.2 Cancers displaying CIN and aneuploidy

The relationship between cancers displaying CIN and the immune system appears to be very complex. Classification of CRCs based on transcriptional profiles showed a subgroup characterized by high numbers of CNAs (43, 152). Despite the high genomic instability, this subgroup appears to be immunologically “cold” in comparison to the other subtypes. A similar trend is observed in EC, where the *TP53* mutant subgroup is characterized by high CNAs, very low immunogenicity and poorer survival outcomes (39, 206). The observation that tumors with low CNAs present a more immune active profile and that high CIN/aneuploid tumors exhibit features of immune exclusion has been made in preclinical models (207) and other cancer types (208). Nevertheless, the reasons for this phenomenon remain to be fully understood.

An interesting study by William and colleagues compared the immune differences between pre-cancerous lesions and later stages of cancer development in HPV^-^ head and neck cancers with major risk SCNAs (209). They found that loss of chromosome 3p, 9p or 17p in pre-cancerous lesions is associated with increased CD3+ T cell infiltration, being trisomy and tetrasomy in chromosome 7 the most strongly associated with overall immune cell density. Interestingly, in later stages of cancer development, the same SCNAs were associated with reduced T cell infiltration, lower cytotoxic activity, and poorer prognosis (209). Furthermore, analysis using TCGA has shown that aneuploid tumors show more features of immune evasion in comparison to non-aneuploid tumors (59, 210). This data demonstrates that CNAs drive a transition from an immune dense to an immune evasive microenvironment over time, but it also suggests they can elicit an immune response, at least in the early stages of tumorigenesis. Subsequent research has shown that acute induction of CIN and/or aneuploidy increases the immunogenicity of tumor cells *in vitro* and *in vivo*, whereas chronic CIN and the resulting aneuploidy ultimately leads to immune evasion, which will be described in more detail further below.

Here, we describe the mechanisms by which immune cells can recognize cancer cells mainly with acute induction of aneuploidy/CIN.

#### 4.2.1 Immune recognition of CIN/aneuploid cells

**Figure 3:**
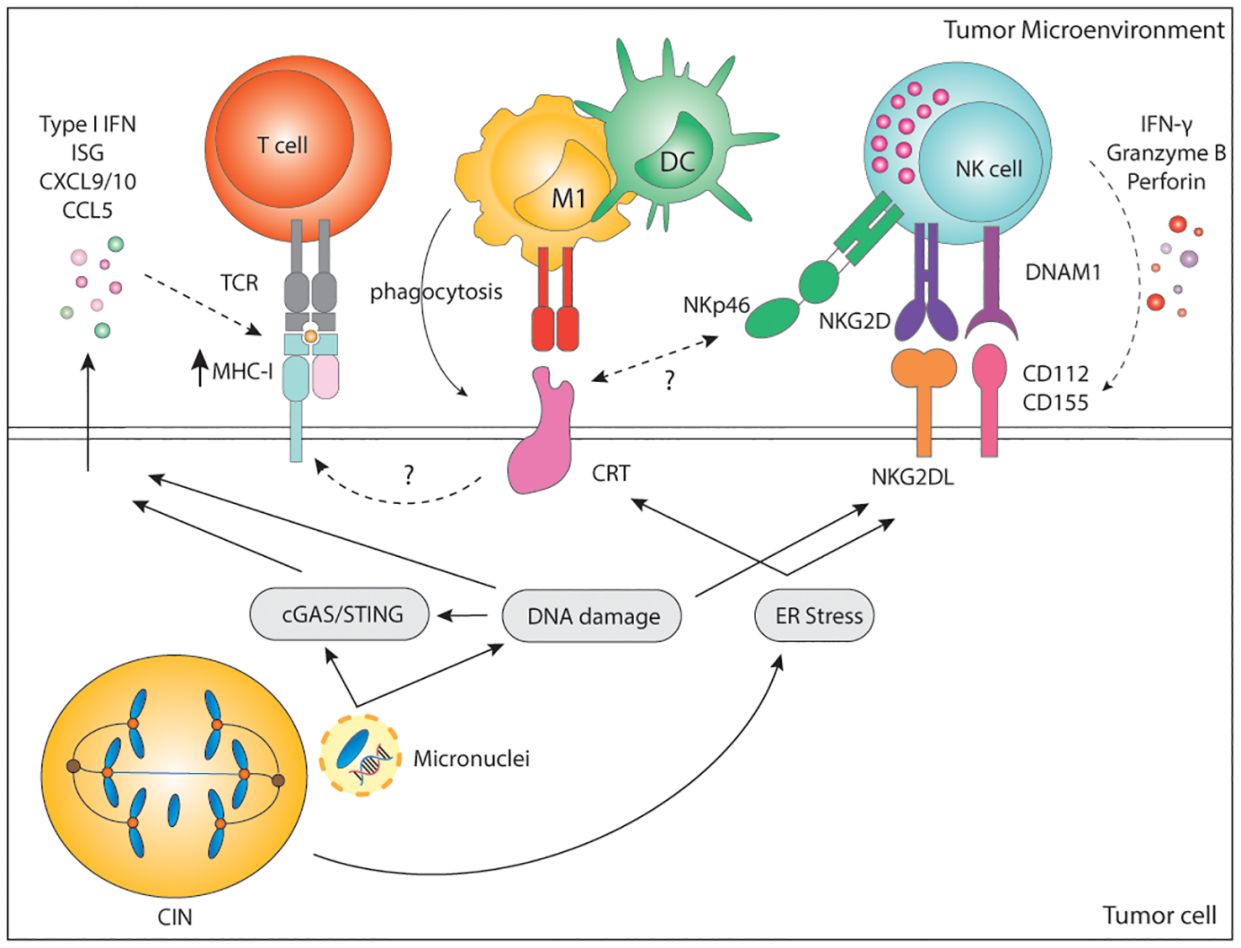
Immune recognition of CIN/aneuploid cells. CIN/aneuploidy trigger a wide range of cellular stressors resulting in the expression of immune activating ligands at the cell surface. CRT is expressed upon ER stress and facilitates phagocytosis by APCs and cytotoxicity by NK cells via the NKp46 receptor. CRT is also known to increase expression of MHC-I for the recognition of stressed cells by CD8+ T cells. In parallel, both DNA damage and ER-stress can upregulate the expression of NKG2D and DNAM-1-ligands, widely known to potently activate NK cells. Finally, secretion of soluble factors by CIN/Aneuploid cells may contribute to immune infiltration, immune activation and increase expression of MHC-I by tumor cells, altogether enhancing their recognition by the immune system. CIN, chromosomal instability; IFN, interferon; ISG, interferon stimulated genes; CXCL, C-X-C motif chemokine ligand; CCL, C-C motif chemokine ligand; MHC, Major histocompatibility complex; TCR, T cell receptor; CD, cluster of differentiation; M1, macrophage type 1; DC, dendritic cell; NK, natural killer; NKG2D, natural killer group 2 D; DNAM-1, DNAX accessory molecule; CRT, calreticulin; ER, endoplasmic reticulum; cGAS, cyclic GMP-AMP synthase; STING, stimulator of interferon response cGAMP interactor.

Besides inflammatory signaling, karyotypic abnormalities can also trigger direct immune recognition mediated by membrane-bound proteins. Hyperploid tumor cells as well as tetraploid *TP53*-/- colon organoids display constitutive endoplasmic reticulum (ER) stress which results in abnormal cell surface exposure of calreticulin (CRT) (211, 212). Interestingly, cells with an extra copy of chromosome 7 did not display ER stress nor abnormal CRT exposure, suggesting that only a major increase in chromosome copy number induces enough ER stress and subsequent CRT surface exposure. Cells with abnormal surface CRT exposure were able to grow in immunocompromised Rag2yc-/- mice, but tumor growth was much slower when injected in immunocompetent mice. Clearance of hyperploid tumors in immunocompetent mice further protected them against rechallenge, indicative of immune memory (211). Indeed, immunosurveillance of hyperploid tumor cells was shown to involve both CD4+ and CD8+ T lymphocytes as well as type I and type II IFN (211) (**Figure 3**). Even though the precise mechanism for CRT redistribution in the cell surface upon high chromosome numbers is not well described, it is long known that CRT serves as an “eat-me signal” and facilitates phagocytosis of stressed cells by APCs, including macrophages and DCs (213, 214), which could explain the findings. In addition, CRT is known to facilitate MHC-I assembly and folding. Thus, higher levels of CRT may also result in higher MHC-I expression by aneuploid cells, increasing their recognition by T cells (215). Finally, a role for Natural Killer (NK) cells cannot be excluded since the widely expressed NKp46 receptor has been recently shown to recognize CRT as a danger-associated signal to eliminate ER-stressed cells (216). CIN and aneuploidy are also known to induce ER stress, which may also induce CRT exposure, enhancing the immunogenicity of such tumor cells (52, 131).

Cultured cell lines with drug-induced aberrant karyotype also show increased immunogenicity *in vitro* as a result of enhanced expression of stress-related DNAM-1 and NKG2D ligands (6, 217). DNAM-1 (CD226) is broadly expressed by various immune cells, including NK and T cells, epithelial and endothelial cells, and through its ligands, CD112 and CD155, mediates cell-to-cell interactions. The activating NKG2D receptor is mainly expressed in NK cells, γδ-T cells, and CD8+ T cells. NKG2D ligands are a highly diversified MHC-I-like family of self-molecules poorly expressed in healthy cells. Binding of the DNAM-1 or NKG2D ligands to their cognate receptor initiates a signaling cascade that results in activation, IFN-γ, and cytokine release by NK cells. Both DNAM-1 ligands and NKG2D ligands are lowly expressed at baseline but can be strongly induced upon viral infection, stress, senescence, and DNA damage (218, 219). Murine and human NKG2D and DNAM-1 ligands are upregulated by genotoxic stress and stalled DNA replication, both known to activate the DNA damage response (4, 220–222). Moreover, sensing of cytosolic DNA in the form of micronuclei has been shown to upregulate the NKG2D ligand RAE1 in murine lymphoma cells in a STING, TBK1, IRF3 and IFN-dependent manner (223). In human melanomas, there is a significant positive correlation between cGAS expression and human NKG2D ligands ULBP1 and ULBP3 (90). This suggests that not only the cGAS/STING pathway is relevant for the production of soluble factors to alert the innate immune system, but it is also involved in the surface expression of danger-associated signals. Nonetheless, the expression and regulation of DNAM-1 and NKG2D ligands upon genomic instability remains to be fully dissected.

In line with previous findings, drug-induced hyperploid cells upregulate CRT but also DNAM-1 and NKG2D ligands. Primary human NK cells cocultured with drug-induced hyperploid cell lines showed increased proliferation, IFN-γ production, and enhanced cytotoxic capacity (217) (**Figure 3**). Santaguida and colleagues showed a similar phenotype in cells with drug-induced complex karyotypes, which *in vitro* were also preferentially killed by the NK92 cell line in comparison to their euploid counterparts (6). NK92 killing of cells with complex karyotypes was further shown to be dependent on both canonical and non-canonical NF-κB but not on type I IFN (224) (**Figure 3**). However, these latter findings remain to be confirmed in a more physiologically relevant context involving primary NK cells. In summary, these data indicate that acute induction of CIN or a high chromosome content can trigger the expression of activating signals for the rapid elimination of cells.

Similarly, MPS1 inhibition-induced aneuploidy of the B16 cell line was shown to increase overall immune infiltration and to favor macrophage polarization to an anti-cancer M1-like phenotype *in vitro* and *in vivo* (225). Further coculture experiments showed that macrophages can suppress the growth of aneuploid/CIN tumor cells *in vitro* (225). However, a comparison of the TME of aneuploid tumors at day 5 and day 10 post-injection into mice revealed a shift from an M1-rich to a pro-tumor M2-like macrophage environment over time (225). Altogether, this data supports the idea that acute induction of CIN/aneuploidy increases the immunogenicity of cells and triggers an immune response that can result in the elimination of the target cells. However, this increase in immunogenicity is reduced over time, ultimately preventing immune recognition and creating a tolerant microenvironment for tumor growth.

## 5 Genomic instability and immune evasion

The prevalence of genomic instability in human cancers indicates that eventually cancer cells and/or their microenvironment become tolerant to genomic damage. Owing to its inflammatory nature, malignant cells with high genomic instability must gain the ability to reduce inflammatory signaling and suppress a productive immune response to fully develop into tumors.

Given the immune-stimulatory effects of cytoplasmic dsDNA and the role of type I IFN in enhancing T cell anti-tumor immunity (100, 226–229), it would be expected that cancer cells with high genomic instability would rewire their response to cytosolic DNA to evade the immune system. Yet, loss of function mutations in either cGAS or STING account for less than 1% of all cancer types (230), potentially explained by the tumor-promoting role of this pathway. The fact that cytosolic DNA can activate the NF-κB cascade without initiating type I IFN indicates that downstream alterations of cGAS/STING signaling are preferred and might favor cancer cells (62). Indeed, dysregulation of the IFN sensing and signaling pathways are well-known immune evasion mechanism across cancer types and correlates with poor response and/or resistance to immunotherapies (231–233). Another common mechanism of cancer immune evasion is downregulation or complete loss of antigen presentation in MHC-I, which impairs CD8+ T cell-mediated immune recognition and thus correlates with poor response to ICB (234). As CD8+ T cells rely on antigen presentation, cancer cells with deficient MHC-I expression remain undetected to CD8+ T cells. Increased expression of inhibitory immune-checkpoint ligands, downregulation of danger-associated signals, secretion of anti-inflammatory factors, or modulation of the TME are other mechanisms to circumvent immune surveillance. These mechanisms are not mutually exclusive and, in most cases, tightly connected. Below, we summarize the main mechanisms of immune evasion described specifically in cancers MMR defects and displaying CIN/aneuploidy.

### 5.1 Immune escape mechanisms in MMRd

Despite having comparable TMB, the immunogenicity and immune infiltration of MMRd tumors can be heterogeneous (34, 235, 236). Accumulating evidence describes a spectrum of immune infiltration in MMRd tumors ranging from robust infiltration of cytotoxic immune cells referred as “hot” tumors, to tumors with none or low density of immune cells, termed “cold” (236). In fact, MMRd with low immune scores or “cold” had a similar immune phenotype to MMRp tumors. Genomics and transcriptomics analysis revealed a subgroup of MMRd CRC displaying low immune and cytotoxic scores characterized by KRAS mutations, Wnt/Notch activation, bigger size, distant metastasis, and early recurrence (235). Furthermore, even though ICB responses in MMRd cancer patients are remarkably positive, they are also heterogeneous. For instance, locally advanced MMRd rectal or colorectal patients achieve nearly 100% complete response rates to anti-PD-1 (237) or anti-PD-1/anti-CTLA-4 treatment respectively (238). In contrast, two doses of anti-PD-1 in local MMRd EC patients yielded major pathological responses in 2/10 patients (239). In the recurrence setting, treatment of MMRd/MSI-H EC patients with anti-PD-1 resulted in complete responses in 12% of the patients and partial responses in 46% of the patients (156). Similarly, a different study reported 48% of objective responses to anti-PD-1 in advanced MMRd EC (240) and comparable results have been shown for metastatic CRC (10, 13) and other non-CRC MMRd tumors (11). These data indicate that between 30 and 60% of patients do not respond to current immunotherapy strategies, suggesting the presence of tumor-intrinsic and extrinsic immune evasion mechanisms that occur during early tumorigenesis and persist through cancer development, ultimately limiting anti-tumor immunity and response to immunotherapy.

#### 5.1.1 Disruption of antigen presentation

**Figure 4:**
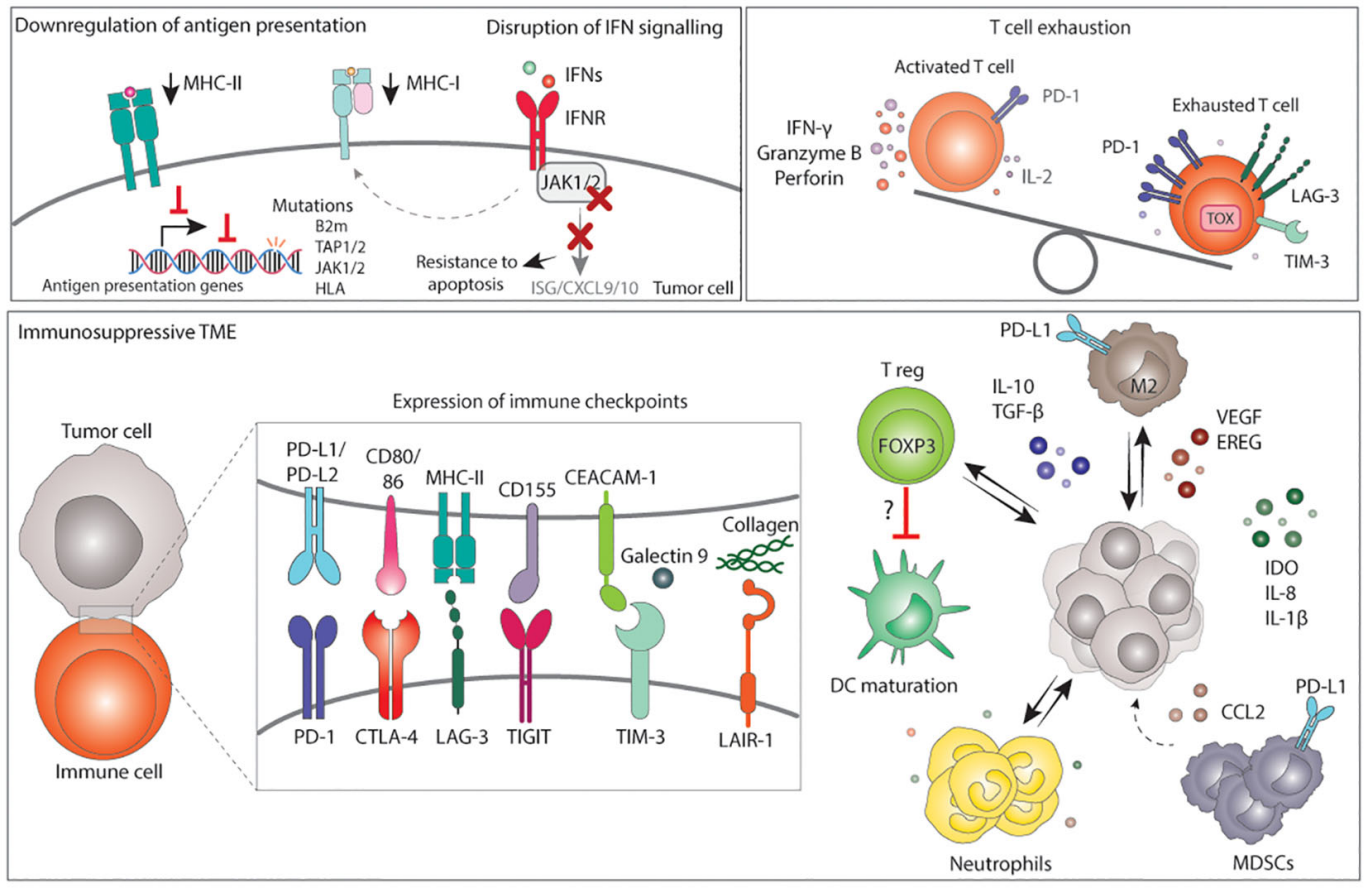
Mechanisms of immune evasion in MMRd tumors. Immune evasion occurs from the selection of clones with more immune evasion features upon an immune selective pressure or immunotherapy. Tumor cells with low MHC-I and/or MHC-II are preferentially selected for. Loss or downregulation of antigen presenting complexes can occur via mutations in antigen presentation genes, mutations in regulators of their transcription, or via disruption of the IFN signalling. Disruption of the IFN signalling generally occurs via mutations in JAK1/2 which in turn result in lower MHC-I expression, lower expression of ISG, CXCL9 and CXCL10, and resistance to apoptosis. Next to tumor cell intrinsic mechanisms, an immunosuppressive TME can also drive immune evasion. The TME of MMRd tumors often displays high expression of immune checkpoint ligands that, together with chronic stimulation, result in T cell exhaustion. In addition, the TME may have high infiltration of M2 macrophages, T reg, neutrophils and MDSCs expressing various immunosuppressive factors, which may also contribute to lower DC maturation, lower T cell activation and a microenvironment that suppress cytotoxic responses, ultimately favoring tumor growth. MHC, major histocompatibility complex; IFN, interferons; IFNR, interferon receptor; B2m, B-2-microglobulin; TAP, transporter associated with antigen presenting; JAK, janus-kinase; HLA, human leukocyte antigen; ISG, interferon stimulated gene; CXCL, C-X-C motif chemokine ligand; PD-1, programmed death 1; IL-2, interleukin 2; LAG-3, lymphocyte-activation gene 3; TIM-3, T-cell immunoglobulin and mucin-domain containing-3; PD-L, programmed death ligand; CD, cluster of differentiation; CEACAM, carcinoembryonic antigen-related cell adhesion molecule; CTLA-4, cytotoxic T lymphocyte-associated protein 4; TIGIT,: T cell immunoreceptor with Ig and ITIM domains; LAIR-1, leukocyte associated immunoglobulin like receptor 1; DC, dendritic cells; T reg, regulatory T cell; FOXP3, Forkhead Box P3; M2, macrophage type 2; TGF-β, transforming growth factor β; VEGF, vascular endothelial growth factor; EREG, epiregulin; IDO, Indoleamine 2,3-Dioxygenase; CCL2, C-C motif chemokine Ligand 2; MDSCs, Myeloid-derived suppressor cells.

Antigen presentation plays a pivotal role in CD8-mediated anti-cancer immunity. Due to enhanced TMB and subsequent neoantigen generation, antigen presentation might be more relevant in establishing an inflammatory microenvironment in MMRd cancers than in other tumor types, increasing the selection pressure to its inactivation (241). Therefore, MMRd cancers often harbor mutations in genes involved in antigen processing and MHC-I complex assembly. Mutations are most commonly found in *TAP1* and *TAP2* genes, which are involved in antigen processing; *B2M*, which is a scaffold protein necessary for the assembly of the MHC-I complex at the cell surface; and human leukocyte antigen I (*HLA-I*) genes, encoding for proteins part of the MHC-I complex (34, 153, 156, 158, 163, 241–247). Notably, the majority of these mutations result in loss of MHC-I function (246). In addition, regulators of HLA gene expression, *NLRC5*, and *RFX5*, are mutated in a significant number of MMRd CRC, leading to decreased expression of MHC-I in tumor cells (242, 245) (**Figure 4**). Although disruptions in antigen presentation have been traditionally considered a mechanism of primary and acquired resistance to ICB across cancer types (234), recent evidence has shown that it is not always the case and that specific immunotherapy combinations can overcome loss of MHC-I (185, 188, 248–250), particularly in MMRd tumors (180, 181, 251, 252). Besides MHC-I, between 20-30% of MMRd colorectal carcinomas show negative or low expression of MHC-II (246, 253, 254), which is used to present antigens to CD4+ T cells. The fact that MHC-II is lost in a proportion of MMRd tumors indicates a selection pressure towards reducing the presentation of antigens to CD4+ T cells (**Figure 4**). Overall, disruption of direct antigen presentation from tumors to immune cells in both MHC-I and MHC-II context is a main mechanism of immune evasion in MMRd tumors.

#### 5.1.2 Alterations in IFN sensing and signaling

Binding of IFNs to their receptor triggers a JAK/STAT-mediated signaling cascade that results in the inhibition of tumor growth and promotes apoptosis. Moreover, IFN signaling increases the expression of MHC-I at the cell surface. Prior studies across multiple contexts have shown that defective IFN signaling protects tumors from IFN-mediated apoptosis, reduced MHC-I expression and reduced CD8+ T cell killing, and is, therefore, a key mechanism of immune evasion across tumors (233, 255). Loss-of-function *JAK1* frameshift mutations are enriched in endometrial and stomach MMRd tumors, are associated with downregulation of the IFN response, lower infiltration of immune cells, limited anti-tumor immunity, and reduced response to ICB (31, 32, 244, 256, 257) (**Figure 4**). Conversely, in a cohort of MMRd CRC, *JAK1* loss-of-function mutations were positively associated with better patient outcome (258) and EC MMRd patients with *JAK1* mutations benefited from anti-PD-1 treatment (156). These opposing results extend to other cancer types and *in vivo* studies (259, 260), which suggest that loss of IFN signaling sensitizes tumor cells to lymphocyte-mediated cell killing and, more broadly, to immunotherapy. Several factors could contribute to these contradictory results. For instance, these studies do not consider the degree of genomic instability and its consequences, the baseline level of IFN signaling in tumor cells, and the tissue type. In conclusion, alterations in IFN sensing and signaling impact tumor development and response to immunotherapy in a context dependent manner, highlighting the complexity of the relationship between IFN signaling and cancer immunity, reviewed elsewhere (261).

#### 5.1.3 Immunosuppressive TME

The colonic mucosa of LS patients (with and without carcinoma) is enriched in exhausted CD8+ T cells, FOXP3+ T reg (262), and high levels of the checkpoint molecules PD-L1 and LAG-3, suggesting an early immunosuppressive TME and an early compromised immune surveillance (167). In fact, MSI-high tumors have a higher density of FOXP3+ T reg cells compared to MSS (263, 264). Interestingly, a recent study also found an inverse correlation between FOXP3+ T reg cells and mature CD208+ DCs, suggesting local immune evasion by impaired DC maturation mediated by T reg (264).

MMRd/MSI tumors are also characterized by high expression of the PD-1/PD-L1 molecules in comparison to MMRp (154, 155, 168). Histological analysis revealed that checkpoint ligands such as PD-L1 are not only expressed by tumor cells but also by other immune cells (154, 155), including myeloid cells at the invasive margin (168) (**Figure 4**). IFNs, among other inflammatory factors, play a role in increasing PD-L1 expression. Thus, the presence of IFNs in the TME could potentially account for the elevated levels of PD-L1 observed in multiple cell types. In fact, MSI CRCs show the highest expression of PD-L1 and PD-L2 genes among the CRC subtypes (152).

Side-by-side comparison of MMRd and MMRp tumors using RNA-sequencing and protein analysis have shown increased expression of other immunosuppressive ligands and molecules such as PD-L2, CTLA-4, LAG-3, TIM-3, TIGIT, LAIR-1, IDO, IL-10, TGF-β, IL-8 and IL-1β in MMRd tumors (103, 152, 153, 168, 170, 187, 265). In line with this, T cells derived from MMRd tumors express high levels of PD-1 and LAG-3 and were enriched in gene programs associated with exhaustion such as TOX (170)(**Figure 4**). Immunosuppressive macrophages and monocytes in MMRd tumors have been described. In fact, in CRCs these cells have been shown to express high levels of tumor-promoting factors (VEGF, EREG) and immunosuppressive molecules (IL-10, TGF-β). In EC, MMRd tumors with an inflamed or “hot” microenvironment contain higher number of T reg cells and M2-like macrophages in comparison to “cold” MMRd tumors (236). Preclinical models have shown that tumor-associated neutrophils can also contribute to immune evasion and anti-PD-1 resistance in MMRd cancers (266). Other studies have reported increased CCL2 expression in MMRd tumors, a chemokine involved in recruitment of immunosuppressive myeloid derived suppressor cells (MDSCs) (152).

Additionally, chronic inflammation can facilitate tumor progression and compromise response to therapy (267). For instance, local inflammatory hubs between T cells, myeloid and malignant cells were specifically found in MMRd but not in MMRp CRC. Such hubs contained exhausted T cells in close proximity with myeloid and malignant cells both expressing ISG gene programs and inhibitory IDO1 and CD38, indicating a negative feedback loop potentially driven by inflammatory signaling (170). In a different study, local inflammatory conditions were recently shown to correlate with poorer response to anti-PD1 in MSI-high CRC (268). In this study, neutrophil-mediated local inflammation suppressed T cell activation possibly through the CD80/86-CTLA-4 axis. Altogether, this suggests that the strongly inflamed microenvironment driven by defective MMR may also increase the presence of self-tolerance mechanisms to restrain immune responses, which may indirectly favor tumor growth (**Figure 4**).

### 5.2 Immune escape mechanisms in CIN/aneuploid tumors

Aneuploidy and CIN correlate with immune evasion, worsened prognosis, and reduced response to immunotherapy in many cancer types (59, 269–272). In comparison to cancers with low CNAs, highly aneuploid tumors are characterized by reduced inflammatory signatures, reduced gene expression of cytotoxic lymphocytes, including T and NK cells, and lower ratios of CD8/T reg and anti-tumor M1/pro-tumor M2 macrophages, indicative of a more immunosuppressive TME (59, 61, 152). This is also observed in a genetically engineered mouse model of EMl4-Alk-driven lung adenocarcinoma. Here, the induction of a CIN phenotype by *Mad2* overexpression increased tumor burden, promoted recruitment of pro-tumor M2 macrophages and impaired CD8+ T cell infiltration and NK cell function in comparison to non-CIN tumors (60).

Immune evasion in tumors displaying CIN is likely to be achieved by the generation of high karyotype heterogeneity and the preferential selection of clones with lower immunogenicity or higher immunosuppressive function upon selective pressure from the immune system. In fact, recurrent patterns of loss or amplification of specific chromosome regions containing known immune regulators have been attributed to reduced immunogenicity and immune evasion in aneuploid tumors (247). Below we summarize the main mechanisms of immune evasion described in cancers with high CIN/aneuploidy.

#### 5.2.1 Dysregulation of the IFN sensing pathway

The most common homozygous deletion across cancer types involves chromosome 9p21.3 (55, 273), which eliminates the cell cycle inhibitor *CDKN2A/B* as well as a type I *IFN* gene cluster. Loss of the 9p21 locus has been widely associated with reduced JAK/STAT, TNFα, and NF-κB signatures, as well as with reduced expression of CXCL9 and CXCL10 chemokines, reduced CD3+ and CD8+ T cell infiltration, and, importantly, with poor prognosis and increased resistance to ICB in several cancer types (209, 233, 273, 274). Genetically engineered cancer cell lines with deletions of the 9p21 chromosome were shown to form more aggressive and metastatic tumors *in vivo* in comparison to cell lines with shorter 9p21 deletions that do not include the *IFN* cluster (275). Mechanistically, type I IFN, largely through secretion of IFN-ϵ by tumor cells, enhances antigen presentation by APCs and generates more functional CD8+ T cells leading to immune control (275). In addition, codeletions with the 9p24.1 locus, containing *JAK2* and PD-1 ligands *CD274* (PD-L1) and *PDCD1LG2* (PD-L2), have been observed. Loss of *JAK2* also contributes to reducing IFN signaling. In fact, 9p24.1 losses were associated with an immune cold phenotype with low CD8+ T cell infiltration and reduced response to anti-PD-1 in head and neck cancer (276).

**Figure 5:**
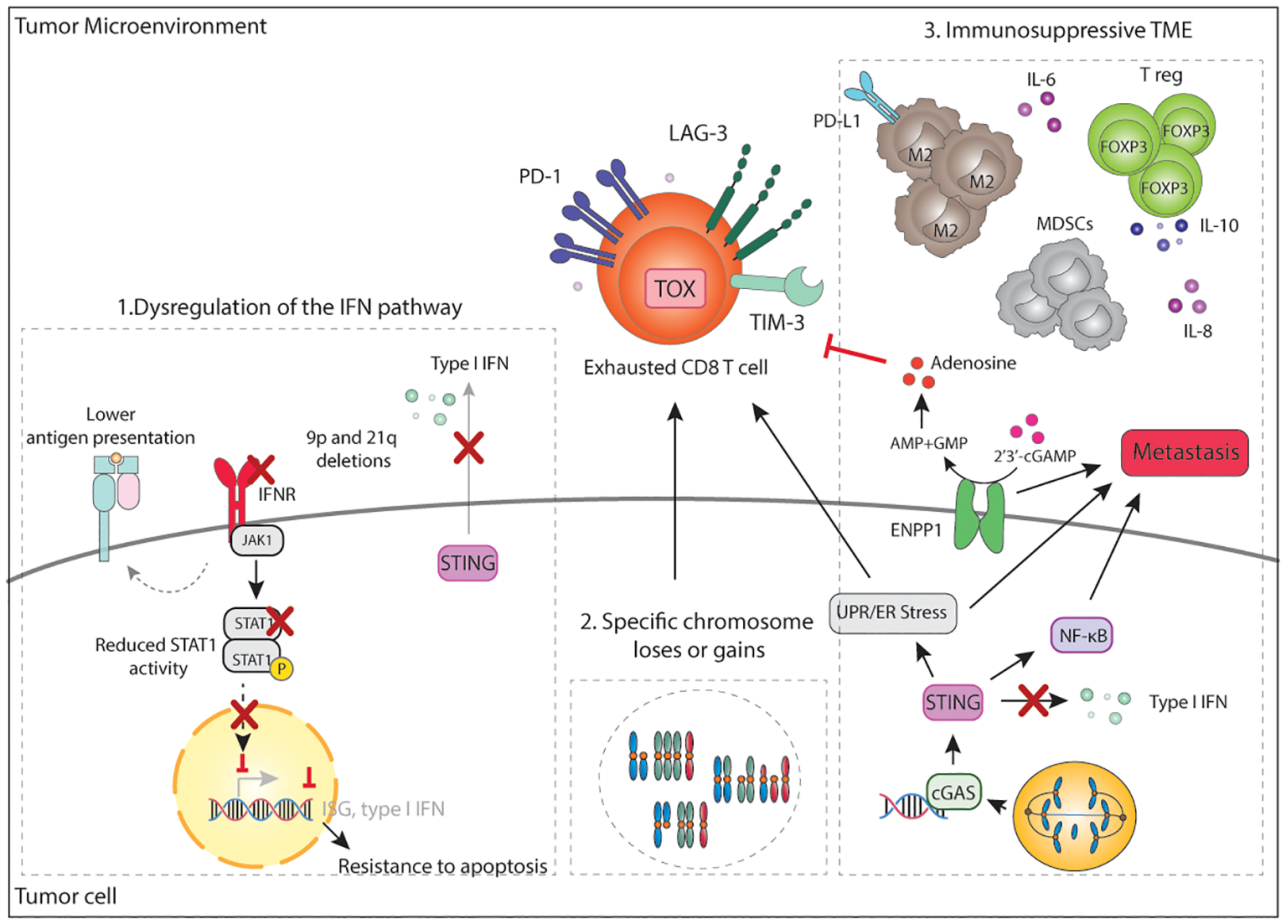
Mechanisms of immune evasion in CIN/aneuploid tumors. A main mechanism of immune evasion in CIN/aneuploid tumors is dysregulation of the IFN pathway. This can occur in multiple ways including 1) loss of the 9p21 arm, which deletes a IFN gene cluster or loss of 9p24 with deletes JAK2, 2) loss of the 21q arm which deletes the IFN receptors, 3) reduced STAT1 activity, 4) chronic stimulation of STING which consequently eschews inflammatory signalling towards NF-κB, while downregulating IFN-signalling. Loss of IFN signalling may also result in decrease MHC-I expression, decrease expression of ISG, CXCL9/10 and resistance to apoptosis. In parallel, increase NF-κB signalling promotes pro-survival signals and initiation of metastasis. CIN tumors also overexpress ENPP1, which eventually leads to the accumulation of adenosine in the TME and inhibition of T cell activity. ER stress and specific chromosome loses or gains can result in an immunosuppressive TME that leads to the accumulation of FOXP3+ T reg, MDSCs, M2 macrophages and immunosuppressive soluble factors, altogether contributing to a dysfunctional and exhausted T cell state. IFN, interferon; IFNR, interferon receptor; ISG, interferon stimulated gene; cGAs; cyclic GMP-AMP synthase; STING, stimulator of interferon genes; PD-1, programmed death 1; LAG-3, lymphocyte-activation gene 3; TIM-3, T-cell immunoglobulin and mucin-domain containing-3; TOX, thymocyte selection associated high mobility group box; CD, cluster of differentiation; UPR, unfolded protein response; ER, endoplasmic reticulum; NF-κB, nuclear factor κB; ENPP1, ectonucleotide pyrophosphatase/phosphodiesterase 1; AMP: adenosine monophosphate; GMP, guanosine monophosphate; IL, interleukin; PD-L1, programmed death ligand 1; T reg, regulatory T cells; TME, tumor microenvironment.

Because IFN signaling also regulates MHC-I expression in an auto and paracrine manner, dysregulation of the type I IFN signaling might also contribute to lower MHC-I expression on the surface of adjacent aneuploid cancer cells and reduce CD8+ T cell recognition of tumors. Downregulation of IFN signaling, and the antigen presentation machinery was observed during the evolution of high CIN tumors in immunocompetent hosts, but not in immunodeficient (61) and human CRCs with high CIN scores generally show lower expression of MHC-I in comparison to other CRC subtypes (152) (**Figure 5**).

It is important to note that besides evasion of the immune system, loss of IFN sensing has also been linked to pro-survival signals and reduced cell death (277). In a mouse model of renal carcinoma displaying high levels of CIN, 9p21 negative tumors spontaneously lose the 16q chromosome (21q in humans) which contains a region with the IFN receptors (277). In line with these findings, an *in vivo* transposon screen identified inactivation STAT1 as one of the main requirements for CIN hematopoietic tumors to fully develop (210). Both data strongly suggest an evolutionary pressure towards suppression of both type I and type II IFN signaling in tumor cells with high levels of CIN. Analysis of publicly available datasets confirmed that aneuploidy/CIN and IFN signaling are inversely correlated (210). Collectively, these studies indicate that reduced IFN signaling and STAT1 activity favor the survival of aneuploid tumors in multiple ways through tolerance of CIN, evasion of the immune system, and contributing to the acquisition of metastatic potential (**Figure 5**).

#### 5.2.2 Specific chromosome losses or gains

Other chromosome losses have also been related to immune evasion. For instance, loss of 3p14 or 17p13 is associated with reduced CD3+ and CD8+ T cell infiltration and reduced immune cell activation makers, but not with CD68+ cells, a macrophage and monocyte marker (209). Rooney et al. showed that amplification of certain genomic regions such as 8p11 and 17p13 were predicted in tumors with low cytotoxic scores (247). The 8p11 region is next to IDO1 and IDO2 enzymes which deplete extracellular tryptophan, creating an immunosuppressive TME (278), thus being a potential mechanism of immune evasion. High-grade serous ovarian cancers, heavily driven by CNAs, often show deletion of the genomic region chr4q35.2 (containing CXCL10 and IL-15 genes, among others) which in turn correlates with reduced formation of TLSs (279). Whereas the presence of TLSs correlates with longer survival, tumors with 4q loss and therefore less TLSs formation responded, worse to ICB and showed a worse prognosis (279). Bladder cancers with loss of the Y chromosome display increased genomic instability and correlate with worsened response to anti-PD-1 and poorer prognoses. Deletion of the Y chromosome was shown to accelerate tumor growth in immunocompetent but not in immunodeficient mice, indicating that tumors deficient in chromosome Y were evading the adaptive immune system more efficiently (280). Loss of the Y chromosome was shown to create a more immunosuppressive microenvironment with a higher presence of terminally exhausted CD8+ T cells expressing TOX and several immune checkpoints such as CD39, TIM-3 and LAG-3. Furthermore, Y-chromosome-negative tumors were enriched in T reg and inflammatory PD-L1+ macrophages in both mice and humans (280). Together, these results demonstrate that loss of specific chromosome or chromosome regions during tumor evolution contributes to altered immune cell function and response to immunotherapy (**Figure 5**).

#### 5.2.3 Modulation of the TME

Next to gains and loss of chromosome arms, CIN and aneuploidy can also orchestrate an immunosuppressive microenvironment for instance by overexpression of ENPP1, a negative regulator of extracellular cGAMP (207). By degrading extracellular cGAMP, ENPP1 prevents paracrine activation of STING and promotes the accumulation of immunosuppressive adenosine, which are two mechanisms contributing to immune evasion (207). Indeed, using mouse models, ENPP1 was shown to reduce immune infiltration of CD8+ T cells and NK as well as the proportion of PD-1+ CD8+ and CD4+ T cells while decreasing the CD8/T reg ratio, consistent with an immunosuppressive TME. Furthermore, ENPP1 was shown to compromise ICB therapy in mice. In human tumors, not only does the expression of ENPP1 negatively correlate with immune cell infiltration, but it is also associated with metastasis (**Figure 5**).

One of the cellular consequences of CIN and aneuploidy is proteotoxic stress and subsequent unfolded protein response (UPR) and ER stress. CIN has been recently shown to drive metastasis in mouse models by modulating the TME through ER stress and UPR signaling (131). In comparison to CIN-low tumors, CIN-high murine tumors display a heavily immunosuppressive TME characterized by the presence of dysfunctional CD8+ T cells expressing exhaustion markers such as TOX, PD-1, TIM-3, LAG-3 and CTLA-4; pro-tumor M2-like macrophages and granulocytic MDSCs. These effects were shown to be mediated by chronic activation of STING on tumor cells, which rewires the intrinsic inflammatory signaling to promote ER stress/UPR signaling while decreasing pro-inflammatory anti-tumor IFN signaling (131). Indeed, upon STING knock-down in CIN-high tumors the effects on the TME were reversed and CD8+ T cells showed higher expression of IFN-γ, granzyme-B and TCF7, indicating a less exhausted cell state (131). These data align with previous research indicating that dysregulation of the IFN signaling is key for tumors with high levels of CIN/aneuploidy. In agreement with these observations, Xian et al. showed that conditional media from aneuploid cells displaying UPR favor polarization of bone marrow-derived macrophages to a pro-tumor M2 phenotype, which were further shown to inhibit T cell activation *in vitro* (269) (**Figure 5**). Although further research is needed to elucidate the role of macrophages and macrophage polarization in CIN/aneuploid tumors, the secretion of soluble factors by aneuploid cells might highjack macrophages to create an environment that supports the growth of aneuploid tumors.

Importantly, in comparison to responders, anti-PD-1 refractory mesothelioma patients showed increased levels of SCNAs, increased T reg numbers in the tumor, and higher levels of IL-6 and IL-8 in the plasma (271). Altogether, this data suggests that by secretion of soluble factors, CIN and aneuploidy rewire the TME and ultimately succeed in favoring tumor growth, metastasis, and resistance to therapy by evasion and modulation of the TME.

### 5.3 The role of p53 in cancers with high genomic instability

Mounting evidence has shown that mutations in *TP53* alter the inflammatory signaling in tumors and heavily affect the immune landscape (281). Tumors with high genomic instability, including CIN tumors, BRCA1/2 mutant tumors, and, in some cases, MMRd tumors, most often harbor *TP53* loss-of-function mutations. Under normal conditions, activation of p53 following a genomic insult enhances anti-tumor immunity by engaging cGAS/STING signaling (282, 283). Therefore, absence of p53 due to mutations would prevent initiation of cGAS/STING signaling. Furthermore, some p53-mutants have been shown to interfere with TBK1 and prevent IRF3-induced signaling downstream of cGAS/STING, contributing to a decrease in type I IFN (284). In cancer cell lines, mutant p53 has been shown to increase expression of TNF-α that in turn sustains pro-tumor chronic NF-κB activation (285, 286). Thus, mutant p53 may favor pro-tumorigenic cGAS/STING-induced NF-κB via attenuating type I IFN signaling. Indeed, a recent study demonstrated that gain-of-function mutations in p53 leads to genomic instability, activation of the cGAS/STING pathway and subsequent non-canonical-NFκB while attenuating IFN signaling (287). This resulted in reduced numbers of tumor-infiltrating T cells, granzyme B+ cells and DCs (287), overall leading to immune evasion and enhanced tumor growth.

Similarly, tumor-specific loss of p53 delayed tumor rejection in a cell-extrinsic manner by inducing CXCL11, CXCL1, CXCL5, CCL3, and M-CSF expression to promote the recruitment of MDSCs and T reg, which were found to attenuate both CD4+ and CD8+ T cell activation (288). In particular, CD4+ and CD8+ T cells from p53-null tumor-bearing mice produced less TNF-α, IFN-γ and IL-2. Similar phenotypes were observed in a genetically engineered murine MMRd lung cancer model with high TMB. Here, deletion of p53 resulted in decreased numbers of CD3+ and CD8+ T cells, a decreased CD8+/T reg ratio, and increased numbers of macrophages, potentially due to increased CCL2 expression by tumor cells. Furthermore, p53 deficient tumors exhibited lower levels of MHC-I and therefore impaired T cell function, which could be rescued by genetic or pharmacological induction of p53 or by stimulation of the cGAS/STING pathway. Importantly, deletion of p53 was shown to drive resistance to ICB in murine tumors and to correlate with worse responses in lung patients treated with anti-PD-1 (146). Similarly, alterations in other oncogenes such as MYC amplification and KRAS mutations have also been linked to enhanced immune evasion features in tumors with high genomic instability (147, 289–292). Collectively, activation of oncogenes or inactivation of tumor suppressors have implications that extend beyond being an oncogenic driver as factors that modulate the inflammatory phenotype, the TME, and the anti-tumor immune response.

### 5.4 Intratumor heterogeneity

Finally, a main factor contributing to immune escape and resistance to ICB in tumors with high genomic instability is intratumor heterogeneity (ITH). ITH refers to the diversity of genetic and phenotypic characteristics of cancer cells within a single tumor. The processes underlying ITH allow individual cancer cells to continually adapt and fosters the acquisition of new features that can contribute to immune evasion and resistance to therapies (293). Some studies have pointed out that sub-clonal neoantigens that are only present in a minority of tumor cells, fail to elicit productive T cell responses. As a result, the ability of the immune system to effectively target and eliminate constantly changing tumor cell populations is severely compromised. In fact, ITH is associated with decreased T cell infiltration and poor survival, whereas a more homogenous tumor composition is generally predictive of response to ICB (177, 294).

## 6 Conclusions and outlook

In this review we discussed the multifaceted interplay between tumor cells with high genomic instability and the immune system, involving various inflammatory signaling pathways and triggering a wide range of cellular responses.

Activation of the cGAS/STING pathway initiates a complex molecular network involving pro- and anti-inflammatory signals. Which pathways are induced upon cGAS/STING engagement appears to be highly dependent on the type of genomic damage, the source of immunostimulatory DNA/RNA, and its dynamics. While MMRd- and HRD-induced cGAS/STING mostly results in the expression of anti-tumor CCL5, CXCL10 and type I IFN; tumors displaying high levels of CIN seem to direct the cGAS/STING response towards NF-κB while attenuating type I IFN signaling, which ultimately promotes pro-metastatic signals and limits anti-tumor immunity. Understanding the balancing act between pro- and anti-tumor inflammatory signaling upon cGAS/STING activation is likely to point the way forward in therapeutic manipulation of this pathway. Furthermore, it is important to shed light on the role of other molecular players involved in sensing self-DNA/RNA, such as the RIG-I/MAVS pathway.

It is also crucial to understand the immunogenic make up of each genomic unstable cancer subtype. In the context of MMRd, there is a strong association between TMB, T cell infiltration, a pro-inflammatory cytokine milieu and favorable outcomes (295). Nevertheless, the immune microenvironment and the response to immunotherapy in MMRd tumors can be highly variable, which underscores the importance of understanding other key determinants of tumor immunogenicity and/or immune evasion. For instance, recent findings suggest that the molecular mechanism of MMRd (germline mutation vs MLH1 promoter hypermethylation) not only affects the TMB but also shapes the cellular composition and functional status of the circulating immune cells and the response to anti-PD-1 (150, 156). Similarly, the immunogenicity and the response to immunotherapy of breast cancers seem to be different between tumors with a *BRCA1* or a *BRCA2* mutation (115, 116, 296). The tissue type also plays an important role: while MMRd ECs and CRCs exhibit exceptional responses to ICB, brain and pancreatic MMRd have limited benefit from current immunotherapies (11). Moreover, affected MS loci are similar between tumors within the same tissue type, but substantially vary between tissues, indicating preferential tissue-specific molecular events (32). Likewise, in the context of immune evasion, human MMRd ECs are enriched in *JAK1/2* mutations, with less frequent mutations in the antigen-presenting machinery; whereas MMRd CRCs show opposite patterns, with frequent mutations in genes for antigen presentation and rarely display alterations in the IFN pathway (32, 297). These observations implicate additional factors that influence the TME and anti-tumor immunity beyond simply the MMR (or HR) status (148).

Conversely, despite initially increasing the immunogenicity of cultured cells and premalignant lesions, CIN/aneuploidy are generally associated with low immune scores and poor response to immunotherapy in cancer. The reasons for this phenomenon remain largely unclear. Firstly, although genomic rearrangements can cause frameshift mutations, whether CIN generates neoantigens that can trigger a productive and sustained T cell response remains a matter of debate. Secondly, recognition of CIN/aneuploid cells by innate cells and membrane-bound ligand-receptor interactions has been mostly reported *in vitro* and data in relevant immunocompetent mouse models and human tumors is scarce. Furthermore, CIN/aneuploid tumors seem to generate a highly immune-excluded and immunosuppressive TME generally characterized by the presence of T reg and M2-macrophages, which limit the access and reactivation of cytotoxic immune cells. Altogether, insights into which aspects of CIN and aneuploidy can activate an immune response and how this response is selectively suppressed during tumor evolution is crucial to identifying therapeutic vulnerabilities in cancers with high CIN/aneuploidy.

From a therapeutic perspective, a single therapy or agent alone is unlikely to be the solution to the complexity and heterogeneity of tumors characterized by high genomic instability. Combinatorial DNA damage inducers or DDR inhibitors that can generate or enhance an (acute) pro-inflammatory milieu in tandem with immunotherapies are likely key for successful clinical outcomes. Such strategies have provided encouraging results in preclinical models and are currently being tested in clinical trials (298, 299).

In conclusion, from the work described above, it has become clear that genomic instability and its consequences add a layer of complexity to tumor biology, anti-tumor immunity, and response to (immuno)therapy. A deeper understanding of how genomic instability shapes the immune microenvironment is thus essential to improve the effectiveness of immunotherapies and select those patients most likely to benefit.
