# Supplementary figures and images for: Genomic instability as a driver and suppressor of anti-tumor immunity
Source: Front Immunol. 2024 Oct 11;15:1462496. doi: 10.3389/fimmu.2024.1462496 (PMC11562473; doi:10.3389/fimmu.2024.1462496)

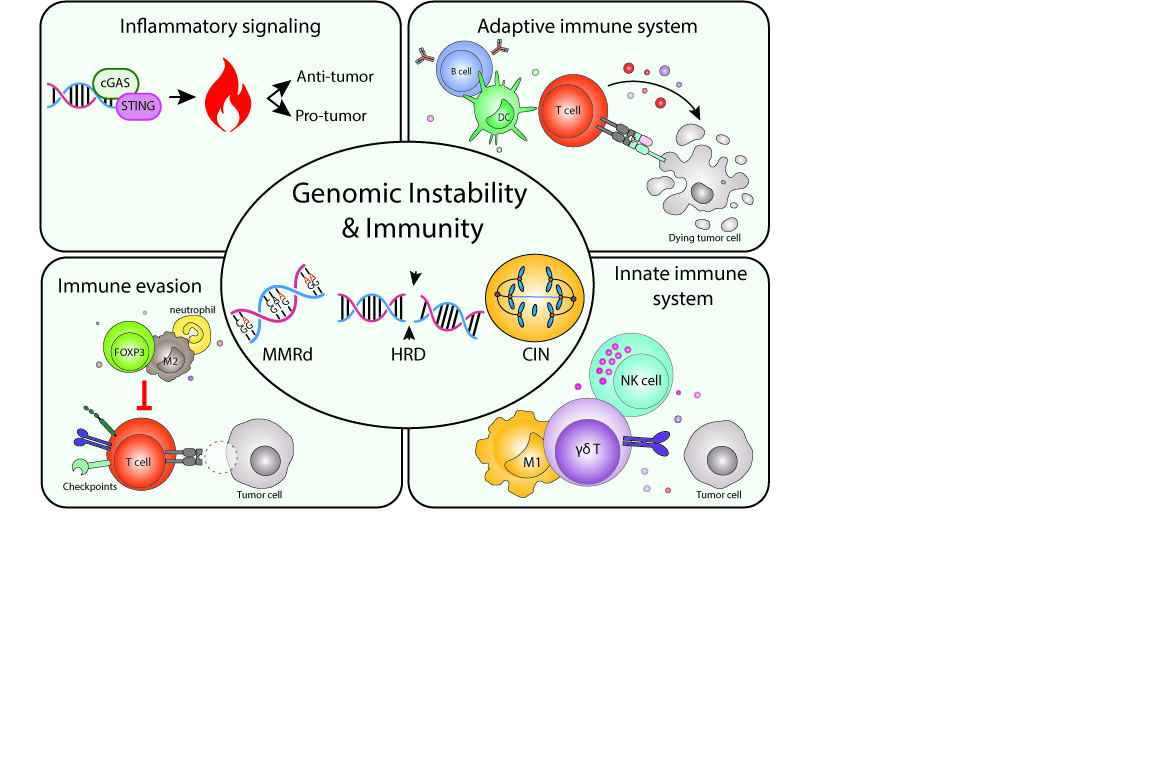

Supplement: Supplementary file 1 [file Image1.tif]
